# Electron microscopy data on irradiation effects in glassy carbon, nuclear graphite, pyrolytic carbon, and carbon fibers

**DOI:** 10.1016/j.dib.2025.111918

**Published:** 2025-07-24

**Authors:** J. David Arregui-Mena, Takaaki Koyanagi, David A. Cullen, Michael J. Zachman, Yan-Ru Lin, Kyle Everett, Sabrina Gonzalez-Calzada, Phillip D. Edmondson, Tyler J. Gerczak, Yutai Katoh, Nidia C. Gallego

**Affiliations:** a Materials Science and Technology Division, Oak Ridge, Tennessee, USA Oak Ridge National Laboratory, Oak Ridge, TN, USA; b Center for Nanophase Materials Sciences, Oak Ridge National Laboratory, Oak Ridge, TN, USA; c Department of Materials, The University of Manchester, Oxford Road, United Kingdom of Great Britain and Northern Ireland, Manchester M13 9PL, UK; d Nuclear Energy and Fuel Cycle Division, Oak Ridge National Laboratory, Oak Ridge, TN 37831, USA; e Chemical Sciences Division, Oak Ridge National Laboratory, Oak Ridge, TN 37831, USA

**Keywords:** Microscopy, TEM, Carbon materials, Neutron irradiation, Nuclear ceramic materials

## Abstract

Glassy carbon, a monoatomic allotrope of carbon, is a candidate material for components in fission nuclear power systems due to its radiation tolerance. This article presents comprehensive electron microscopy data revealing the effects of neutron and electron irradiation on glassy carbon. For comparison, additional data are provided for pyrolytic graphite and carbon fibers, materials that exhibit similar structural behavior under irradiation. *In situ* electron irradiation experiments further illustrate the real-time microstructural evolution of glassy carbon during exposure. The dataset is organized into five parts: (1) transmission electron microscopy (TEM) micrographs of as-received and neutron-irradiated glassy carbon; (2) TEM micrographs of neutron-irradiated graphite; (3) TEM micrographs of unirradiated and irradiated carbon–carbon composites; (4) TEM micrographs of pyrolytic carbon specimens in both conditions; (5) scanning transmission electron microscopy (STEM) micrographs of as-received and neutron-irradiated glassy carbon and (6) *in situ* electron irradiation data of a glassy carbon particle. These datasets provide valuable insights into radiation-induced structural changes in carbon-based materials relevant to nuclear applications.

Specifications Table

**Table:** 

Subject	Engineering & Materials science
Specific subject area	*This dataset consists of electron micrographs characterizing the effects of neutron irradiation on four materials relevant to nuclear applications: glassy carbon, nuclear graphite, pyrolytic carbon, and carbon–carbon composites.*
Type of data	Raw images are provided in .tif format and consist of transmission electron microscopy micrographs. The image folders are labeled according to their corresponding figure in this article and irradiation conditions.
Data collection	The TEM bright-field data was collected using a JEOL 2100F transmission electron microscope operated at 80 kV in bright-field mode. Bright-field TEM images and electron energy loss spectroscopy (EELS) data from the *in situ* experiment were acquired using a JEOL 2100F operating at 200 kV and equipped with a Gatan Image Filter (GIF) Quantum SE spectrometer. The STEM data was acquired using an aberration corrected JEOL NEOARM STEM operated at an accelerating voltage of 80 kV.
Data source location	Oak Ridge National Laboratory; Oak Ridge, Tennessee
Data accessibility	Repository name: *Electron Microscopy Data on Irradiation Effects in Glassy Carbon, Nuclear Graphite, Pyrolytic Carbon, and Carbon Fibers* https://data.mendeley.com/datasets/vgch54bf6d/1 Data identification number: 10.17632/vgch54bf6d.1
Related research article	*This research is associated with the following publication:* J.D. Arregui-Mena, T. Koyanagi, D.A. Cullen, M.J. Zachman, Y.R. Lin, P.D. Edmondson, Y. Katoh, Comprehensive characterization of the irradiation effects of glassy carbon, *Acta Materialia* 281 (2024) 120,441.

## 1 Value of the Data

• The compiled transmission electron microscopy (TEM) data provide critical insights into the microstructural evolution of various carbon-based materials.
• The TEM micrographs serve as a comprehensive database that researchers can use to compare against other irradiation conditions.
• The data inform the selection and engineering of carbon-based materials for nuclear applications by elucidating how different materials respond to irradiation.

## 2 Background

**Fig. 1:**
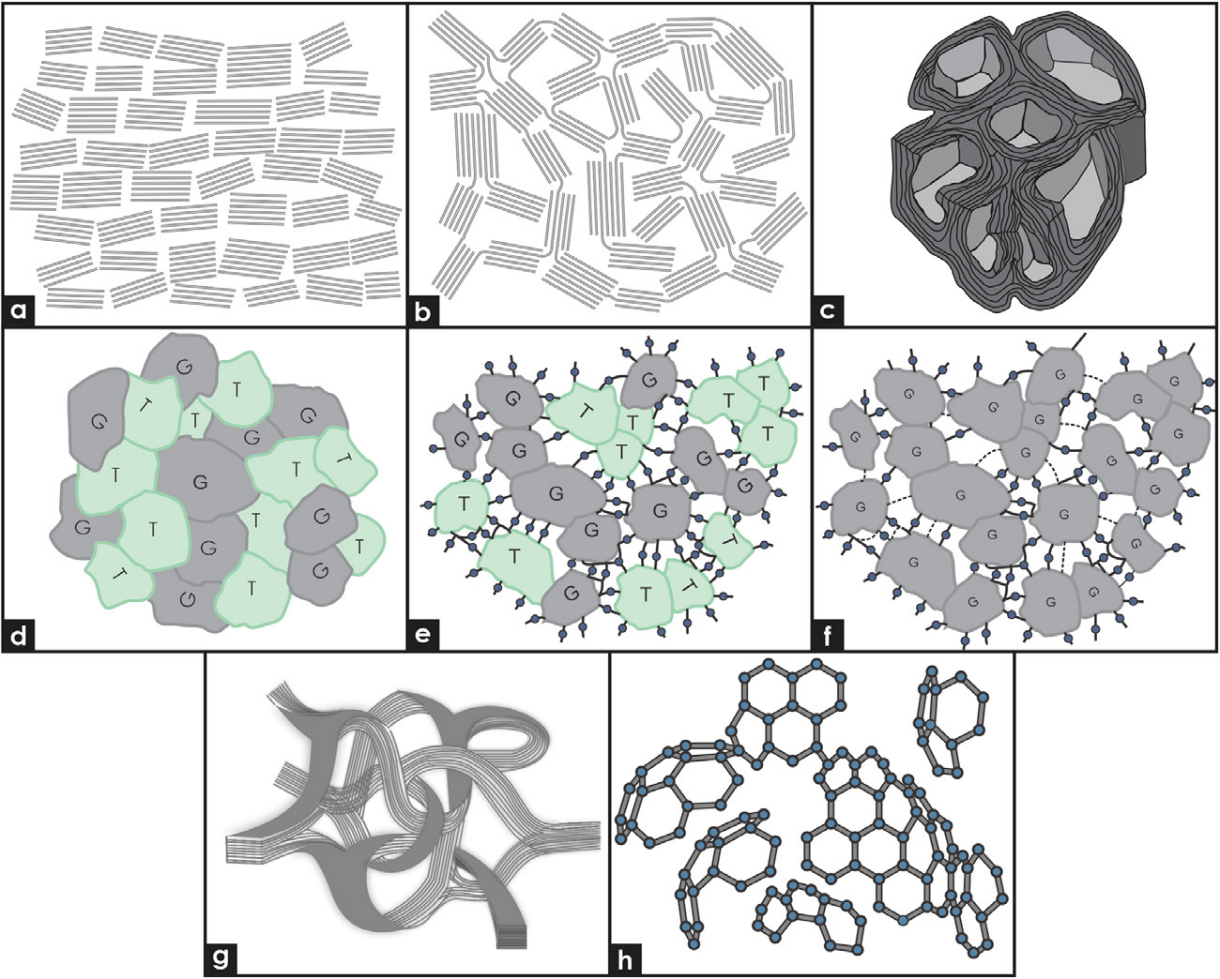
Schematics of models of the development of glassy carbon. Franklin’s depiction of (a) graphitizable and (b) non-graphitizable amorphous carbons [8]. (c) Model of microtexture of glassy carbon treated at high temperatures based on another study [9]. (d) Original glassy carbon model of Noda and Inagaki [10]: G: graphitic cluster, T: tetrahedral cluster. (e) Kakinoki model: G: graphitic cluster, T: tetrahedral cluster, **:** oxygen bridge [11]. (f) Modified model of Noda and Inagaki based on Szeluga et al. [12]: G: graphite, **:** oxygen bridge, ····: other bonds. (g) Jenkins and Kawamura model [13] formed by a network of ribbon stacks. (h) Fullerene-like structure of glassy carbon by Harris based on another study [14].

Glassy carbon is a non-graphitizable allotrope of carbon characterized by a microstructure consisting of short-range atomic arrangements that resemble graphitic domains. Several structural models have been proposed to better understand its microstructure and mechanical properties (Fig. 1). This article compiles microstructural data from neutron-irradiated samples of glassy carbon and other carbon-based materials such as nuclear graphite, carbon–carbon composites, and pyrolytic carbon. Specific regions known to be particularly susceptible to neutron irradiation were selected to highlight the pronounced structural changes observed across all materials—changes that, to some extent, resemble the behavior of glassy carbon. These data are critical because they demonstrate that certain phases across various carbon materials exhibit behaviors like those of glassy carbon under neutron irradiation. Comparative analysis of transmission electron microscopy (TEM) data provides deeper insights into how different carbon materials respond to neutron irradiation.

The data presented in this manuscript are associated with the research study [1] and complement several datasets reported therein. The data presented in this manuscript are also intended for comparison with previously published TEM studies [[2], [3], [4], [5], [6], [7]]. Specifically, this manuscript includes TEM data and extends ongoing efforts to build a comprehensive database of carbon materials relevant to the nuclear industry.

## 3 Data Description

### 3.1 Transmission electron microscopy DATA

**Fig. 2:**
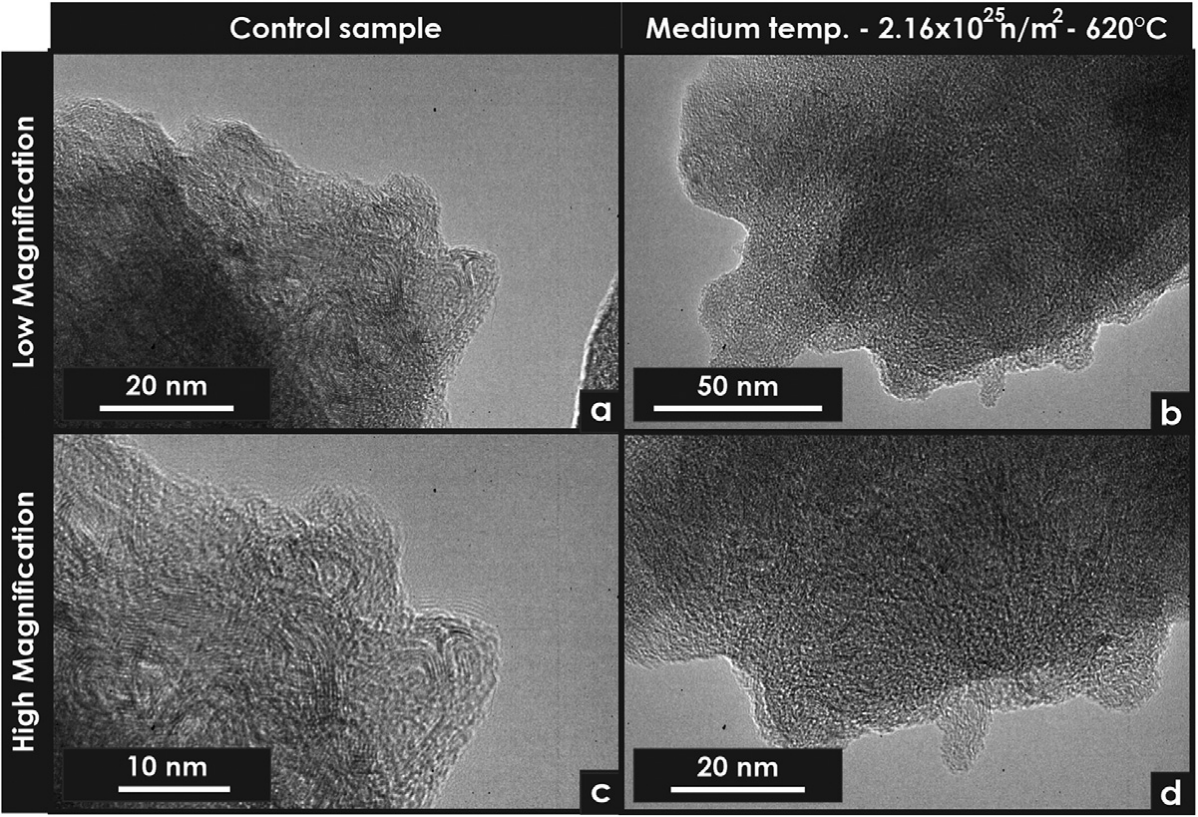
Transmission electron microscopy micrographs of unirradiated and neutron-irradiated glassy carbon specimens. These micrographs were extracted from particles. (a) Low-magnification micrograph of an unirradiated specimen. (b) Low-magnification micrograph of a neutron-irradiated specimen. (c) high-magnification micrograph of an unirradiated specimen. (d) High-magnification micrograph of a neutron-irradiated specimen.

**Fig. 3:**
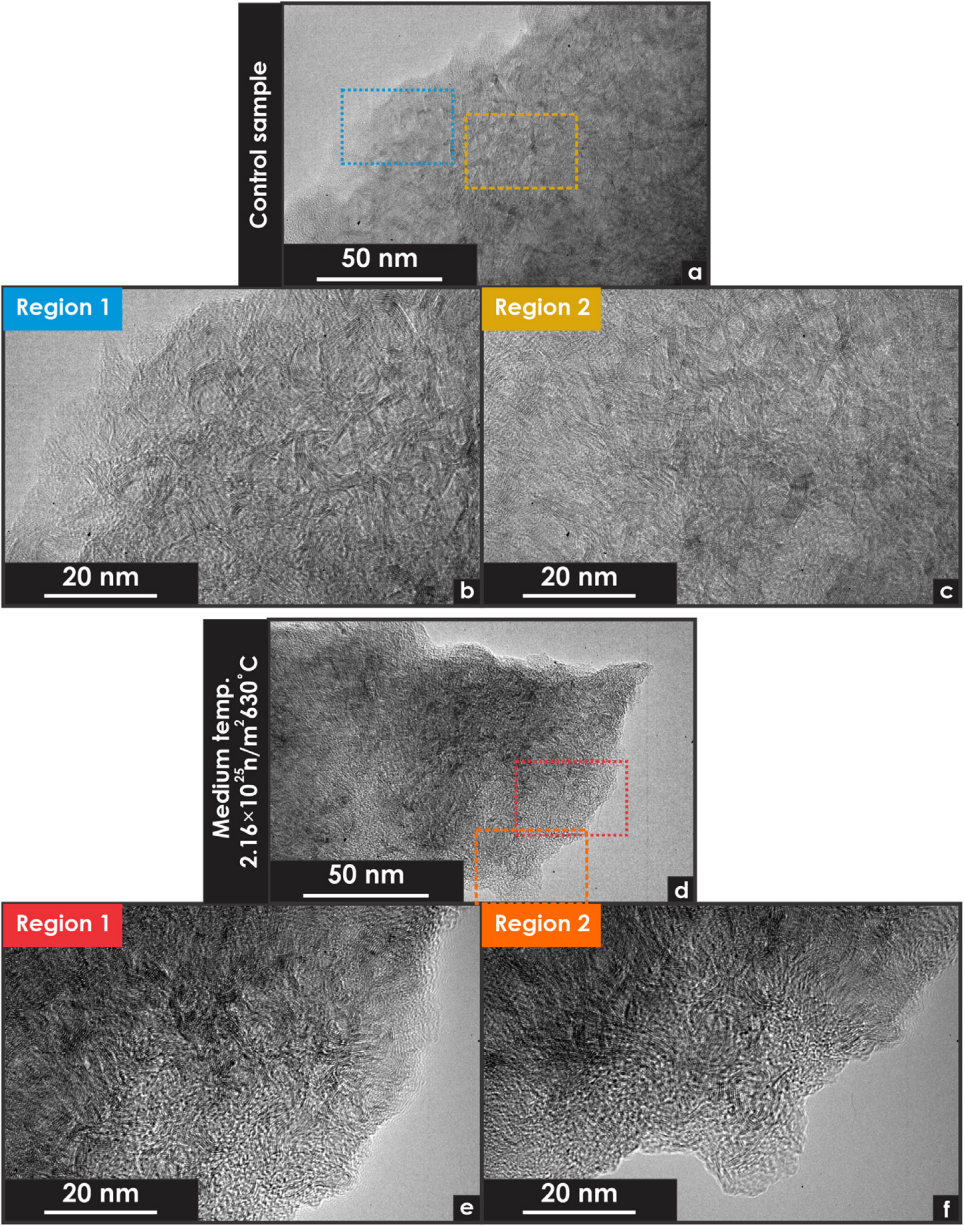
Transmission electron microscopy micrographs of unirradiated and neutron-irradiated glassy carbon specimens. All images were obtained from particle-based samples. (a) Low-magnification micrograph of an unirradiated specimen. (b) High-magnification micrograph of an unirradiated specimen (Region 1). (c) High-magnification micrograph of an unirradiated specimen (Region 2). (d) Low-magnification micrograph of a neutron-irradiated specimen. (e) High-magnification micrograph of a neutron-irradiated specimen (Region 1). (f) High-magnification micrograph of a neutron-irradiated specimen (Region 2).

**Fig. 4:**
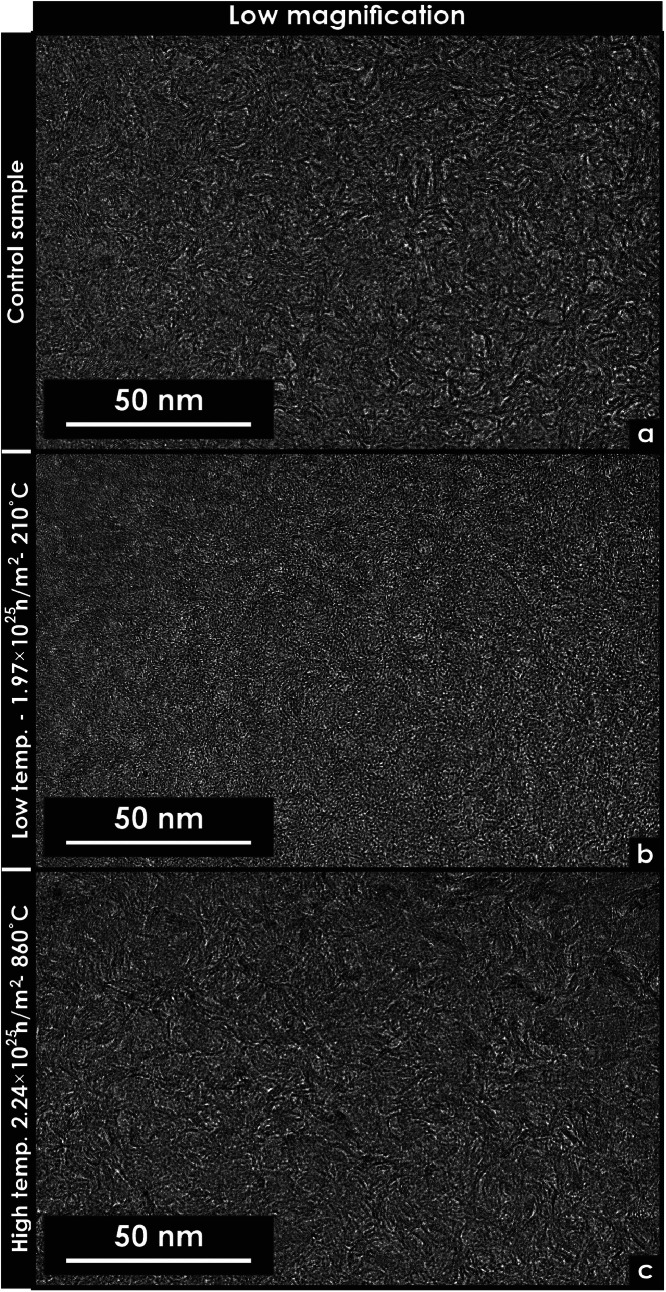
Transmission electron microscopy micrographs of unirradiated and neutron-irradiated glassy carbon specimens. These micrographs were extracted using focused ion beam techniques. (a) Low-magnification micrograph of an unirradiated specimen. (b) Low-magnification micrograph of a specimen irradiated at low temperature. (c) Low-magnification micrograph of a specimen irradiated at high temperature.

**Fig. 5:**
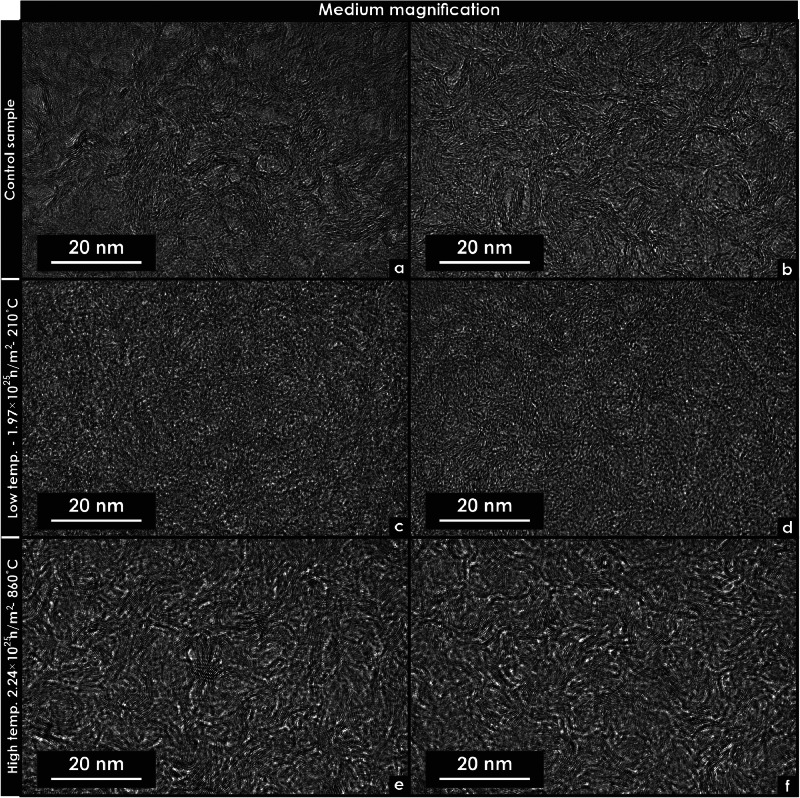
Transmission electron microscopy micrographs of unirradiated and neutron-irradiated glassy carbon specimens. These micrographs were extracted using focused ion beam techniques. (a) Medium-magnification micrograph of an unirradiated specimen in Region 1. (b) Medium-magnification micrograph of an unirradiated specimen in Region 2. (c) Medium-magnification micrograph of a specimen irradiated at low temperature (Region 1). (d) Medium-magnification micrograph of a specimen irradiated at low temperature (Region 2). *(*e) Medium-magnification micrograph of a specimen irradiated at high temperature (Region 1). (f) Medium-magnification micrograph of a specimen irradiated at high temperature (Region 2).

**Fig. 6:**
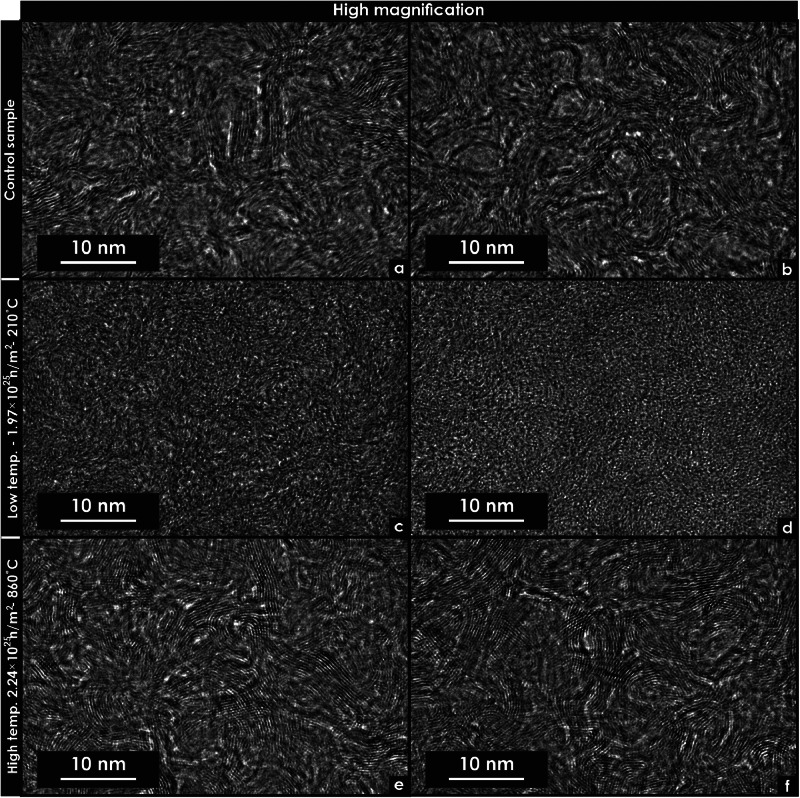
Transmission electron microscopy micrographs of unirradiated and neutron-irradiated glassy carbon specimens. These micrographs were extracted using focused ion beam techniques. (a) High-magnification micrograph of an unirradiated specimen in Region 1. (b) High-magnification micrograph of an unirradiated specimen in Region 2. (c) High-magnification micrograph of a specimen irradiated at low temperature (Region 1). (d) High-magnification micrograph of a specimen irradiated at low temperature (Region 2). (e) High-magnification micrograph of a specimen irradiated at high temperature (Region 1). (f) High-magnification micrograph of a specimen irradiated at high temperature (Region 2).

**Fig. 7:**
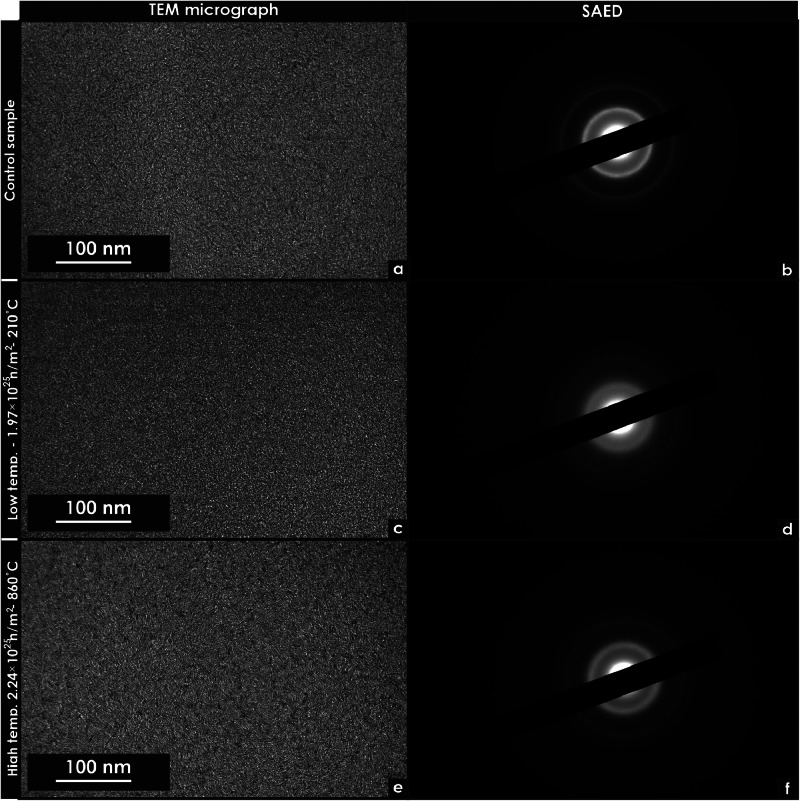
Transmission electron microscopy micrographs of unirradiated and neutron irradiated glassy carbon specimens. These micrographs were extracted using focused ion beam techniques. (a) Low-magnification micrograph of an unirradiated specimen. (b) Selected area electron diffraction (SAED) pattern of the low-magnification micrograph of an unirradiated specimen. (c) Low magnification micrograph of a specimen irradiated at low temperature. (d) SAED pattern of the low-magnification micrograph of a specimen irradiated at low temperature. (e) Low-magnification micrograph of a specimen irradiated at high temperature. (f) SAED pattern of a specimen irradiated at high temperature (Region 2).

**Fig. 8:**
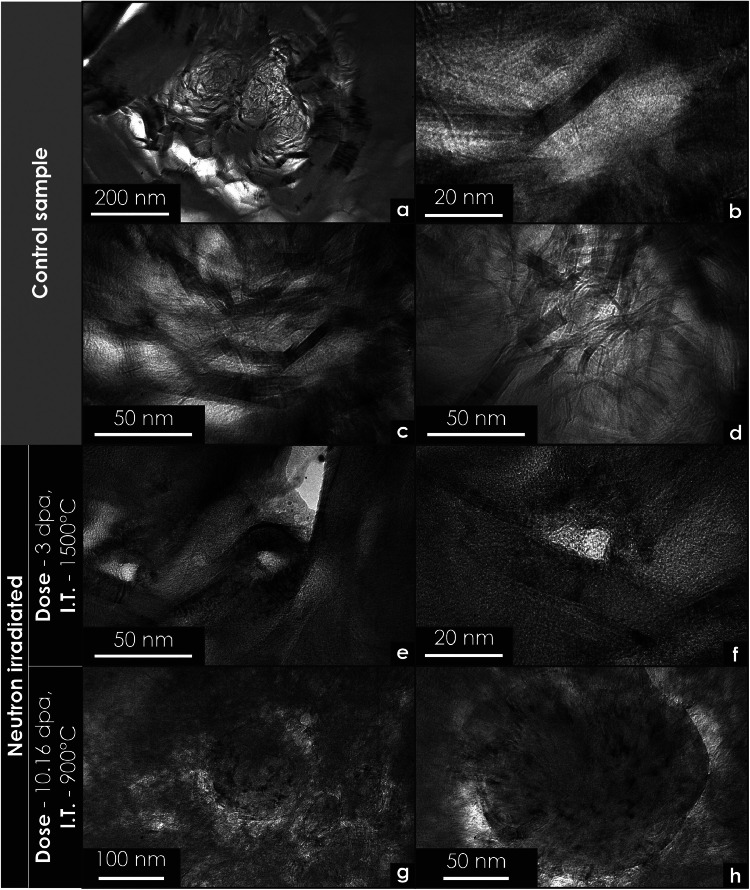
Transmission electron microscopy micrographs of unirradiated and neutron-irradiated PCEA graphite specimens. All specimens were prepared using focused ion beam techniques. (a) Morphology of a quinoline-insoluble particle in an unirradiated control specimen. (b–d) Examples of highly oriented strands located near pores. (e–f) Circular formations observed near pores following irradiation. (g–h) Large carbon onion structures identified in irradiated specimens: these are possibly densified quinoline particles.

**Fig. 9:**
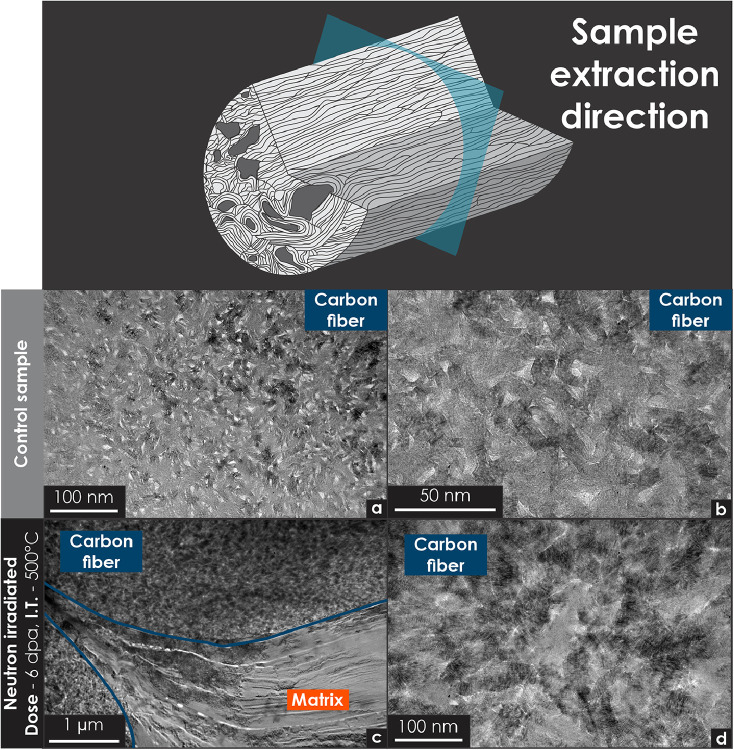
Transmission electron microscopy micrographs of unirradiated and neutron-irradiated carbon–carbon composite specimens (FMI-222). The sampling location, oriented perpendicular to the fiber axis, is indicated in the diagram at the top of the illustration. All specimens were prepared using focused ion beam techniques. (a) Low-magnification micrograph showing the cross-sectional morphology of an unirradiated (control) sample. (b) High-magnification micrograph detailing the cross-sectional features of the control sample. (c) Low-magnification micrograph of an irradiated sample, revealing two fibers and the surrounding matrix. (d) High-magnification micrograph showing postirradiation texture of the carbon fiber.

**Fig. 10:**
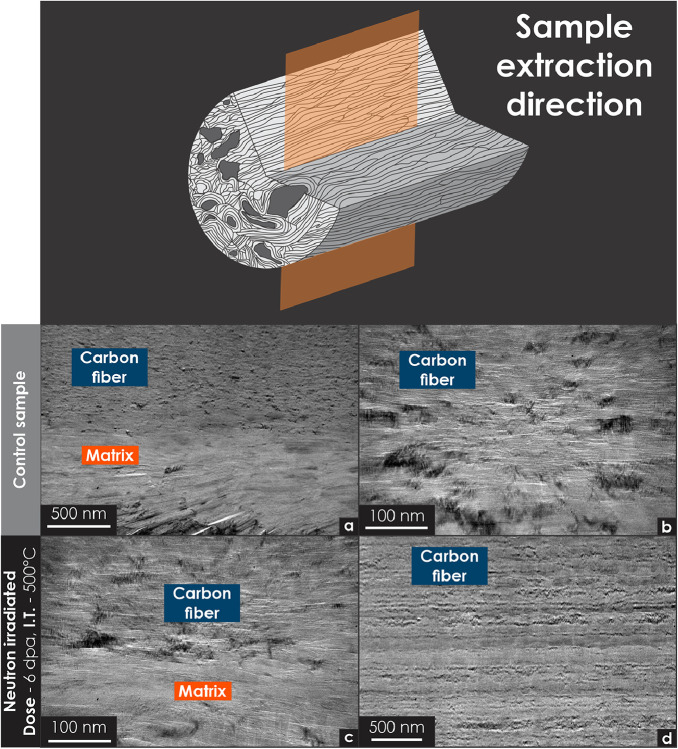
Transmission electron microscopy micrographs of unirradiated and neutron-irradiated carbon–carbon composite specimens (FMI-222). The sampling location, oriented parallel to the fiber axis, is indicated in the diagram at the top of the illustration. All specimens were prepared using focused ion beam techniques. (a) Low-magnification micrograph showing the morphology of an unirradiated (control) sample. (b) High-magnification micrograph detailing the cross-sectional features of the control sample. (c) Low-magnification micrograph of an irradiated sample, illustrating the altered fiber texture after irradiation. (d) High-magnification micrograph highlighting the fiber texture after irradiation.

**Fig. 11:**
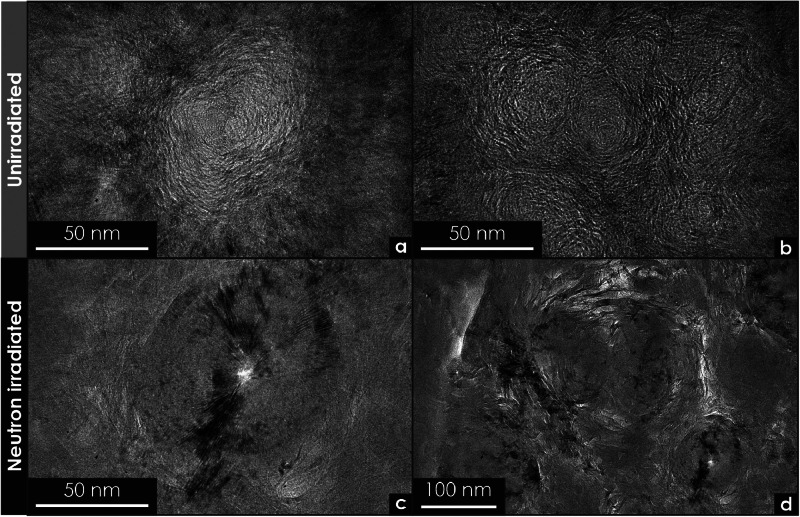
Transmission electron microscopy micrographs of unirradiated and neutron-irradiated pyrolytic carbon extracted from tristructural-isotropic (TRISO) fuel particles. (a) Low-magnification micrograph showing the morphology of an unirradiated (control) sample. (b) High-magnification micrograph highlighting common microstructural features observed in the control sample. (c) Low-magnification micrograph of an irradiated sample illustrating the changes in pyrolytic carbon texture following irradiation. (d) High-magnification micrograph detailing the microstructural evolution of the pyrolytic carbon after irradiation.

Transmission electron microscopy (TEM) data were acquired from particles of glassy carbon (Fig. 2, Fig. 3) and from various carbon-based materials; most micrographs were obtained from samples prepared using focused ion beam (FIB) techniques (Fig. 4, Fig. 5, Fig. 6, Fig. 7, Fig. 8, Fig. 9, Fig. 10, Fig. 11). Micrographs obtained from particles offer additional insight into the microstructure of glassy carbon because this sample preparation method introduces fewer defects and contains thinner regions where fewer domains overlap.

Figs. 4–6 display the morphology of glassy carbon at multiple magnifications. Fig. 6 shows the microstructure of glassy carbon along with the corresponding selected area electron diffraction (SAED) pattern from the same region. Figs. 8a to d highlight unirradiated areas adjacent to quinoline-insoluble particles or pores filled with graphitic strands, resembling those observed in glassy carbon. By contrast, Figs. 8e to h illustrate the effects of neutron irradiation in similar regions, showing irradiation-induced morphological changes.

Fig. 9 presents the morphology of a carbon fiber specimen extracted perpendicular to the fiber axis. Figs. 9a and b show the microstructure of an unirradiated specimen, whereas Figs. 9c and d illustrate irradiation-induced effects, particularly fiber densification. Figs. 9a and b exhibit structural features that resemble those observed in the glassy carbon micrographs presented in Figs. 4 through 8.

Fig. 10 displays the morphology of a carbon fiber specimen extracted parallel to the fiber axis. Figs. 10a and b present the cross-sectional features of the unirradiated fiber, whereas Figs. 10c and d reveal postirradiation changes, including pore closure and structural densification.

Fig. 11 compares the morphology of pyrolytic carbon control specimens and neutron-irradiated specimens used in the inner pyrolytic carbon layer of tristructural-isotropic (TRISO) coated particles. Figs. 11a and b show representative micrographs from unirradiated control samples, and Figs. 11c and d depict the microstructural evolution following irradiation.

## 4 Scanning Transmission Electron Microscopy Data

**Fig. 12:**
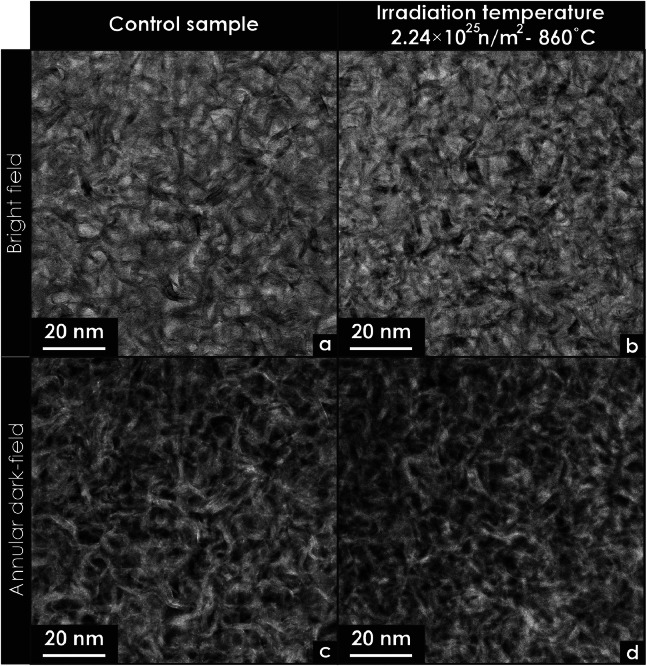
Scanning transmission electron microscopy (STEM) micrographs of unirradiated and neutron-irradiated glassy carbon specimen. (a) Bright field STEM micrograph of an unirradiated specimen. (b) Bright field STEM micrograph of a neutron irradiated specimen at high temperatures, (c) Annular dark-field STEM micrograph of an unirradiated specimen. (b) Annular dark-field STEM micrograph of a neutron irradiated specimen at high temperatures.

Bright-field and annular dark-field scanning transmission electron microscopy (STEM) images were acquired from glassy carbon specimens prepared using FIB techniques (Fig. 12). These images were included to assess and compare porosity in a control sample and a specimen subjected to high-temperature irradiation.

## 5 *In situ* Electron Irradiation of Glassy Carbon

**Fig. 13:**
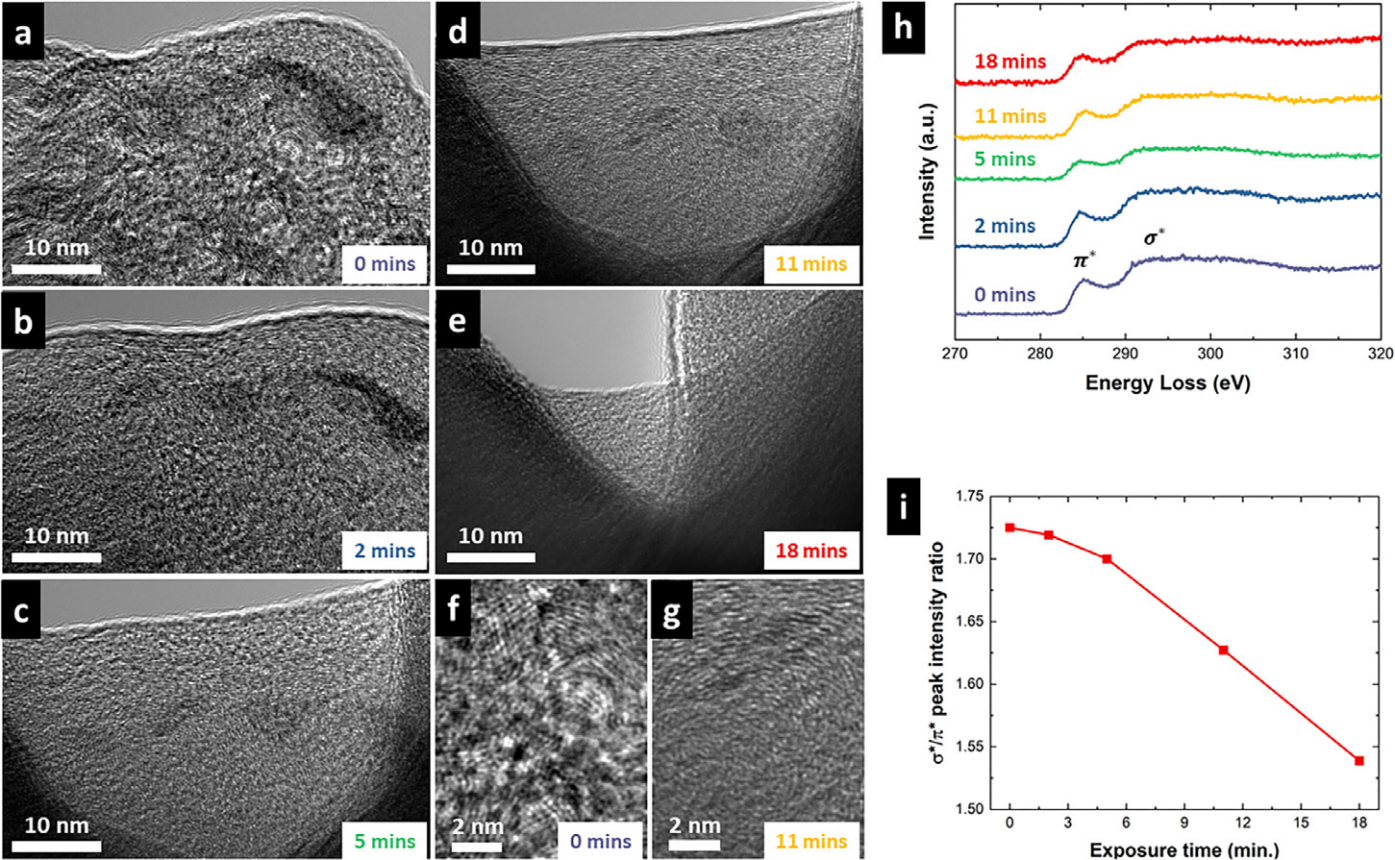
*In situ* electron irradiation experiment on a glassy carbon particle. (a - e) Bright-field TEM micrographs of the same region captured during electron irradiation. These images demonstrate the high mobility of carbon under electron beam exposure. The elapsed time of the experiment is indicated in the lower right corners. (f - g) High-resolution bright-field TEM micrographs showing the texture of glassy carbon before and after irradiation. (h) EELS spectra collected at different time points during the irradiation experiment. (i) Ratio of σ* to π*, illustrating the evolution of the EELS spectra over time.

An additional experiment was conducted to investigate the mobility of carbon domains in glassy carbon under electron irradiation. In these experiments, electron beam damage served as a surrogate mechanism to simulate the movement of graphitic strands in glassy carbon. Bright-field images were taken at various times (Fig. 13a to e). These micrographs show the high mobility of the glassy carbon thin particle section and changes in thickness. In addition, the original and final structures of the irradiated glassy carbon area were documented using high-resolution TEM (Fig. 13f and g). Moreover, electron energy loss spectroscopy (EELS) was performed at different time points to show the evolution of the glassy carbon over time (Fig. 13h). The selected EELS spectrum energy range includes two distinct features: the π* peak near 285 eV and the broader σ* peak around 290 eV, with a plateau between them. The π* peak is typically associated with sp² bonding and results from excitations of 1 s core-level electrons into the antibonding π* state. The σ* peak arises from excitations of carbon 1 s electrons into the antibonding σ* state. As shown in Fig. 13h, irradiation led to a reduction in the σ* peak compared to the initial pre-irradiation spectrum. To better understand these changes, the ratio of σ* to π* was included to show the evolution of the EELS spectra of glassy carbon during the irradiation process (Fig. 13i).

## 6 Experimental Design, Materials and Methods

The prepared glassy carbon powders (Fig. 2) were crushed using a mortar and pestle in distilled water to reduce particle size. For TEM specimen preparation, the ground powder was sonicated in distilled water for 5 min and subsequently deposited onto a lacy carbon-coated grid. All other samples were prepared using FIB techniques, as described in elsewhere [5].

TEM microscopy characterization was conducted using a JEOL 2100F TEM instrument operated at 80 kV to minimize beam-induced damage. Bright-field TEM micrographs were acquired from the various materials; this technique is widely used to resolve fine features in carbon materials ranging from below 10 nm to approximately 1 ´m. A camera length of 20 cm was used to acquire the SAED patterns using an aperture to focus only on the center of the beam.

The specimens used for STEM characterization were conducted using a JEOL NEOARM STEM operated at 80 kV in bright field and annular dark-field modes. The same glassy carbon FIB samples were used for both TEM and STEM imaging.

The glassy carbon samples manufactured by Tokai Carbon Co, LTD were irradiated at the High Flux Isotope Reactor (HFIR) at Oak Ridge National Laboratory. Properties of this glassy carbon are listed in Table 1. Carbon fiber composites (FMI-222) were manufactured by Fiber Materials Inc were also irradiated at HFIR. The graphite specimens, sourced from PCEA—a nuclear-grade graphite manufactured by GrafTech—were irradiated at the Advanced Test Reactor (ATR) at Idaho National Laboratory. The pyrolytic carbon was extracted from TRISO particles fabricated by BWXT used in the AGR-2 irradiation tests conducted at the ATR. The specimens used in this study were extracted from particles subjected to destructive post-irradiation examination at Oak Ridge National Laboratory [15]. The irradiation conditions for the glassy carbon samples and all the other carbon materials are summarized in Table 2.

**Table 1:** Typical properties of Tokai carbon Co, LTD glassy carbon.

Property	Values
Apparent density	1.51 g/cm^3^
Flexural strength	147 MPa
Thermal conductivity	5.8 W/m·K

**Table 2:** Conditions for neutron-irradiated specimens.

ID	Material	Irradiation temperature ( °C)	Fluence (10^25^ n/m^2^)	Dose (dpa)	Specimen preparation method
GL50	Glassy carbon	210	1.97 (*E* > 0.1 MeV)		FIB
GL53	Glassy carbon	630	2.16 (*E* > 0.1 MeV)		Powder
GL64	Glassy carbon	860	2.24 (*E* > 0.1 MeV)		FIB
DW61	Graphite	1500		3.06	FIB
G10B4	Graphite	900		10.16	FIB
FMI	Carbon–carbon composite	500		6	FIB
PyC	Pyrolytic carbon	1287	3.35 (*E* > 0.18 MeV)		FIB

The *in situ* irradiation was performed using a JEOL 2100F transmission electron microscope operating at 200 kV and room temperature that is equipped with a Gatan Image Filter (GIF) Quantum SE spectrometer. An unirradiated glassy carbon specimen was sectioned from the bulk material, crushed, dispersed in an alcohol solution, and mounted on a TEM microgrid. This preparation method promotes the formation of thin regions containing only a few stacked graphitic layers, suitable for high-resolution imaging. Bright-field TEM images were taken at various time intervals to document the microstructural evolution over time and EELS spectra.

## Limitations

Samples prepared using FIB techniques exhibit varying thicknesses, which can lead to overlapping carbon domains in the glassy carbon specimens, thereby limiting certain aspects of image analysis.

## Ethics Statement

The authors confirm that they have read and adhere to the ethical requirements for publication in Data in Brief. This work does not involve human subjects, animal experiments, or data collected from social media platforms.

## CRediT authorship contribution statement

**J. David Arregui-Mena:** Conceptualization, Methodology, Formal analysis, Investigation, Data curation, Writing – original draft. **Takaaki Koyanagi:** Formal analysis, Investigation, Data curation, Resources, Supervision. **David A. Cullen:** Data curation, Funding acquisition, Visualization, Investigation. **Michael J. Zachman:** Data curation, Funding acquisition, Visualization, Investigation. **Yan-Ru Lin:** Formal analysis, Investigation, Methodology, Data curation, Investigation. **Kyle Everett:** Resources, Methodology. **Sabrina Gonzalez-Calzada:** Resources, Methodology. **Phillip D. Edmondson:** Conceptualization, Methodology, Formal analysis. **Tyler J. Gerczak:** Resources, Supervision, Methodology. **Yutai Katoh:** Resources. **Nidia C. Gallego:** Resources.

## Data Availability

Mendeley DataElectron Microscopy Data on Irradiation Effects in Glassy Carbon, Nuclear Graphite, Pyrolytic Carbon, and Carbon Fibers (Original data).
